# Editorial: Materials for mechanotransduction and beyond

**DOI:** 10.3389/fcell.2023.1222957

**Published:** 2023-05-26

**Authors:** Surabhi Sonam, Jenny Malmstrom, Ken-ichoro Kamei, Matthew J. Dalby

**Affiliations:** ^1^ School of Biosciences and Bioengineering, D. Y. Patil International University, Pune, India; ^2^ Department of Chemical and Materials Engineering, University of Auckland, Auckland, New Zealand; ^3^ Programs of Biology and Bioengineering, New York University Abu Dhabi, Abu Dhabi, United Arab Emirates; ^4^ School of Molecular Biosciences, University of Glasgow, Glasgow, United Kingdom

**Keywords:** mechanobiology, surface geometry, GelMA, surface stiffness, micropatterning, 3D structures, biomechanics

Cells actively sense and process the mechanical information in their surroundings to make decisions about growth, motility, and differentiation. They exert forces on their surrounding matrix and adjacent cells via the functions of actin cytoskeleton and adhesion machineries and utilize them to perform crucial cell processes like cell movement and differentiation. Mechanobiology has underpinned many scientific advances in understanding how biophysical and biomechanical cues regulate cell behavior. Such studies have identified both mechanosensitive proteins and specific signaling pathways within the cell that govern the production of proteins necessary for cell-based tissue development and remodelling, or, indeed, regeneration. To understand the cellular mechanoresponses, it is pertinent to develop new cell materials and new tools, which can regulate the mechanical environment presented to the cells and eventually govern cell behavior, including proliferation, differentiation, gene expression, protein synthesis, matrix production, apoptosis, and necrosis of the cells.

This Research Topic includes articles that have generated innovative tools and techniques to generate mechanical stimuli of living cells and tissues. The development of new tools and materials is essential for advancing our understanding of mechanobiology and its role in health and disease.

In the first article, Eren et al. use topographies to grow tenocytes that adapt to surface geometry by remodeling their focal adhesions and actin cytoskeleton. The authors showed that the surface geometry eventually modulated the cell shape and physiology, such as shape-induced differentiation, metabolism, and proliferation. The formation of focal adhesions, actin cytoskeleton, and the forces placed on to the material was shown to be regulated and determined by the geometry of the surface. The article concludes that understanding the mechanisms by which cells adapt to surface geometry can provide insights into the development of tissue engineering and regenerative medicine.

In the second article, Cimmino et al. have developed a novel method for controlling cell adhesion by using a light-responsive azopolymer conjugated with protein micropatterns of varying topography. This innovative approach can be applied to generate complex 3D structures for regenerative medicine and tissue engineering. By using a confocal laser-based technique, the researchers were able to direct cell migration and alter submicrometric topographic patterns. This, in turn, affected cell spreading and shape, and permitted the authors to follow dynamic changes in cell adhesion, cytoskeletal structures, and nucleus conformation which were generated by the alterations in the adhesive properties of the substrate. This approach can be used to investigate mechanotransduction-related events in a dynamic manner, by controlling cell adhesion at both the focal adhesion and cell shape levels.

Stiffness of the substrate is known to modulate the cell migration, division and differentiation. The third article by Chalard et al. discusses the feasibility of patterning the stiffness of GelMA hydrogels with visible light to create *in vitro* models of healthy and fibrotic tissue. GelMA is a biocompatible material that can be precisely tuned for its mechanical properties and is widely used in tissue engineering. This article describes the use of visible light and eosin Y as a photoinitiator to photopattern the mechanical properties of GelMA hydrogels using physical photomasks and projection with a digital micromirror device. The resulting hydrogels had areas of different stiffnesses and stiffness gradients. The article concludes that this method could be used to build *in vitro* models of healthy and fibrotic tissue and to study cellular responses to fibrotic mechanisms.

In the fourth article, Levario-Diaz et al. created 1D micro-nanopatterned integrin ligand surfaces to direct cell movement. The researchers used di-block copolymer micellar nanolithography to create nanoparticles with varying integrin ligand spacing, and observed the effects on cell adhesion and migration, which could have applications in wound healing, tissue formation, and cancer metastasis. The study found that the nanoscale spacing of integrin ligands can be used to direct cell movement, and could be a promising approach for developing cell-instructive biomaterials.

The fifth article Monferrer et al. can be useful for understanding the mechanobiology of neuronal cells by providing a 3D cell culture system that mimics the *in vivo* microenvironment of neuroblastoma tumors. The study incorporated full-length vitronectin in polyethylene glycol hydrogels to create a tunable 3D cell culture system that supports neuroblastoma cell growth and migration. The hydrogel composition was adjusted to mimic different stages of neuroblastoma progression. This system can be used to study the role of various extracellular matrix (ECM) components in neuroblastoma growth and progression. Understanding the role of ECM components in neuroblastoma growth can provide insights into the mechanobiology of neuronal cells and the development of neuroblastoma tumors.

Understanding the mechanical nature of cell systems in 1D to 3D scale is important because it can provide more insights on physiologically relevant environment for cells growth and migration. The articles in this Research Topic have provided multiple different tools and material modification techniques to generate adequate mechanical responses from cell to further understand the process of cell adhesion, migration, tissue growth and disease progression.

